# Analyses of quality of life in cancer drug trials - a review of measurements and analytical choices in post-reimbursement studies

**DOI:** 10.1186/s12885-024-12045-8

**Published:** 2024-03-06

**Authors:** Mikael Svensson, Gabriella Chauca Strand, Carl Bonander, Naimi Johansson, Niklas Jakobsson

**Affiliations:** 1 Department of Pharmaceutical Outcomes & Policy, College of Pharmacy, University of Florida, 1225 Center Dr, Gainesville, FL 32610 USA; 2 School of Public Health & Community Medicine, Institute of Medicine, University of Gothenburg, Gothenburg, Sweden; 3 Centre for Societal Risk Research, Karlstad University, Karlstad, Sweden; 4 University Health Care Research Center, Faculty of Medicine and Health, Orebro University, Orebro, Sweden; 5 Karlstad Business School, Karlstad University, Karlstad, Sweden

**Keywords:** Health-related quality of life, Patient-reported outcomes, Cancer, Randomized controlled trials, Quality assessment

## Abstract

**Objectives:**

For drugs reimbursed with limited evidence of patient benefits, confirmatory evidence of overall survival (OS) and quality of life (QoL) benefits is important. For QoL data to serve as valuable input to patients and decision-makers, it must be measured and analyzed using appropriate methods. We aimed to assess the measurement and analyses of post-reimbursement QoL data for cancer drugs introduced in Swedish healthcare with limited evidence at the time of reimbursement.

**Methods:**

We reviewed any published post-reimbursement trial data on QoL for cancer drugs reimbursed in Sweden between 2010 and 2020 with limited evidence of improvement in QoL and OS benefits at the time of reimbursement. We extracted information on the instruments used, frequency of measurement, extent of missing data, statistical approaches, and the use of pre-registration and study protocols.

**Results:**

Out of 22 drugs satisfying our inclusion criteria, we identified published QoL data for 12 drugs in 22 studies covering multiple cancer types. The most frequently used QoL instruments were EORTC QLQ-C30 and EQ-5D-3/5L. We identified three areas needing improvement in QoL measurement and analysis: (i) motivation for the frequency of measurements, (ii) handling of the substantial missing data problem, and (iii) inclusion and adherence to QoL analyses in clinical trial pre-registration and study protocols.

**Conclusions:**

Our review shows that the measurements and analysis of QoL data in our sample of cancer trials covering drugs initially reimbursed without any confirmed QoL or OS evidence have significant room for improvement. The increasing use of QoL assessments must be accompanied by a stricter adherence to best-practice guidelines to provide valuable input to patients and decision-makers.

**Supplementary Information:**

The online version contains supplementary material available at 10.1186/s12885-024-12045-8.

## Background

Alongside the objective of assessing cancer drugs in terms of Overall Survival (OS) improvements, there is an increasing focus on assessing patient-reported outcomes, such as self-assessed Quality of Life (QoL) [1–4]. Many healthcare jurisdictions also rely on cost-effectiveness analysis with Quality-Adjusted Life-Years (QALYs) as an outcome measure to inform reimbursement and coverage decisions, which requires combining QoL and OS data [5]. Valid measurements and analyses of QoL in cancer trials are fundamental for healthcare decision-makers and patients since such analyses can facilitate well-informed priority-setting and lead to better patient-centered care. Analyses of QoL endpoints in cancer drug treatments are essential in the absence of survival data or when improvements in survival are unlikely.

At the time of regulatory market authorization of cancer drugs, it is frequent that data on OS or QoL confirming clinical benefits is lacking. Market authorization decisions are instead often based on intermediate (surrogate) endpoints such as Progression-Free Survival (PFS) and Response Rates [6–9], which frequently lack validation as predictors of long-term OS and QoL benefits. Following market authorization, many healthcare systems rely on subsequent Health Technology Assessments (HTA) to decide on reimbursement and to inform pricing negotiations [10–12]. The HTAs usually depend on the same pivotal trial data underlying the market authorization for the clinical- and cost-effectiveness modeling, facing challenges with immature OS data and a lack of published QoL data. Therefore, it is in the interest of patients and payers to learn if OS or QoL benefits can be confirmed post-reimbursement based on updated analyses of the pivotal trials or from new controlled or pragmatic trials [13, 14]. In addition, the frequent reimbursements of high-cost cancer drugs based on uncertain surrogate endpoints [15] call for more post-reimbursement studies on patient-relevant outcomes using valid and reliable measures and analytical methods [16–18].

Cancer patients’ quality of life, including health-related QoL, can be measured using generic, cancer-generic, or cancer-indication-specific instruments [19, 20]. Generic instruments that can be applied across health conditions, such as the EQ-5D instrument [21], facilitate comparative analyses of patient benefits across therapeutic areas, which is relevant for decision-makers and horizontal priority setting. Cancer-specific instruments, such as the EORTC QLQ-C30 [22], or even more detailed indication-specific instruments, may capture more granular aspects of QoL, which can be particularly important for clinical decision-making and vertical priority setting. Irrespective of the instruments used, shortcomings have been identified in terms of a lack of defined hypotheses, lack of analyses to account for missing data and multiple hypothesis testing, lack of discussions of clinical significance [23–27], and absent or deficient study protocols [28]. There have been several calls to improve the validity and reliability of QoL assessments in cancer trials by standardization and guidelines on high-quality reporting, including recommendations from the Consolidated Standards of Reporting Trials (CONSORT-PRO) [29] and the Standards in Analyzing Patient-Reported Outcomes and Quality of Life (SISAQOL) Consortium [30]. Despite the increased attention to QoL analyses in cancer trials, no previous study has specifically addressed post-reimbursement follow-ups of drugs approved with initially limited evidence on OS and QoL.

In this study, we aim to assess measurements and analyses of QoL in post-reimbursement studies of cancer drugs to identify potential areas with room for improvement in cancer trial QoL research. Specifically, we analyze cancer drugs introduced in Swedish healthcare with limited evidence, defined as lacking randomized trial data or analysis showing any statistically significant OS or QoL benefits at the time of reimbursement. In Sweden, the Pharmaceutical and Benefits Agency (TLV) decides on the reimbursement of prescription drugs, and a large majority of all cancer drugs with European Medicines Agency (EMA) market authorization receive reimbursement [9]. In a previous study, we identified all reimbursed cancer drugs between 2010 and 2020 that had limited evidence of OS and QoL benefits at the time of reimbursement. We also analyzed how many drugs subsequently verified benefits in post-reimbursement studies [31].

The present study concerns the approaches to measurement and analysis of QoL data used to verify patient-relevant benefits, and our contributions are twofold. First, we analyze the reporting and analytical choices of QoL data specifically for cancer drugs that lacked statistically significant evidence on patient-centered outcomes (QoL and OS) at the time of reimbursement. This addresses to what extent such evidence is generated post-reimbursement to reduce the uncertainty of the benefits of these drugs, and to the best of our knowledge, this has not been the focus in the previous literature. Second, we compare the adherence of reporting and analytical choices of QoL data to what was outlined in clinical trial pre-registrations, study protocols, and statistical analysis plans, and we provide novel results regarding to what extent the analyses can be seen as confirmatory or merely exploratory. The findings from this study can be used to identify areas with room for improvement in the measurement and analysis of QoL data in cancer trials.

## Methods

### Identification of cancer drug indications with post-reimbursement QoL data

We identified prescription cancer drug indications approved by TLV between 2010 and 2020 where the producer claimed a therapeutic benefit compared to the standard of care but where the evidence base for this argument was limited. We defined limited evidence as drugs where no randomized trial data showed statistically significant (*p* < 0.05) OS or QoL benefits in published studies or based on the material submitted to TLV. Instead, the therapeutic benefit supposition was based on improvements in surrogate/intermediate endpoints and/or based on single-arm trials. From a total of 60 reimbursement applications, 46 drug indications were approved by TLV to be included in the Swedish Pharmaceutical Benefits Scheme. Of those 46 drug indications, 22 had limited evidence at the time of a favorable reimbursement decision.

For each of these 22 drug indications, we searched PubMed and Clinicaltrials.gov for any post-reimbursement evidence on QoL or OS from randomized controlled trials (RCT) until September 2022. We used the following search string to identify post-reimbursement studies for each of the 22 drug indications: “active substance name OR drug brand name AND cancer form AND Cochrane Highly Sensitivity Search Strategy for identifying randomized trials” [31]. We included new publications from the original pivotal trial and any other randomized (clinical or pragmatic) trials related to the specific indication for each reimbursement decision. We allowed for the inclusion of studies irrespective of whether the research objective was explicitly outlined to demonstrate clinical benefit based on an apriori hypothesis or if the objective was to conduct exploratory QoL analyses. We have previously published the full details of the search protocol and the results documenting the number of drug indications with any RCT post-reimbursement data on OS or QoL [31]. The search strategy and Flow-diagram are also presented in the Supplementary material. The work in this study was based on publicly accessible information and did not involve individual patient information. No formal ethical approval was therefore required.

### Data extraction and analysis

Two authors (MS and NiJ) independently extracted information using a pre-defined data extraction template. The data extraction template covered basic study information and several established quality indicators of research design and statistical practice: the study and year, cancer type/drug indication, QoL instrument(s) used, the scoring system or tariffs used to summarize QoL data, the frequency of QoL measurements, the statistical analyses of the QoL scores, proportion of missing QoL data, if missing data adjustments were conducted, if a detailed study protocol or statistical analysis plan describing data collection and statistical analyses were published, and if all QoL instruments in the study were listed in the pre-registration on clinicaltrials.gov before the analyses were conducted (before finalizing data collection). Disagreements in the extracted data were resolved by discussion, and the data is presented using descriptive statistics.

## Results

We identified post-reimbursement RCT data on QoL in 22 studies covering 12 of the 22 included drug indications (Supplementary material, Figure S1 & Table S1). Of the 22 studies, five were published between 2010 and 2016 and 17 between 2017 and 2022. The indications covered by the studies included the following cancers: lung, breast, kidney, ovarian, leukemia, and melanoma. A total of 4 of the 22 studies reported statistically significant (*p* < 0.05) QoL benefits (statistically significant QoL benefits for the reimbursed drug compared to the control arm). In contrast, the other 18 studies reported findings where the QoL data were not statistically significantly different between the reimbursed drug and the comparator or that the reimbursed drug had statistically significant detrimental QoL effects. Table 1 summarizes the studies in terms of the QoL measurement and analysis.

**Table 1:** Summary of the QoL assessment characteristics

Characteristics of QoL assessment	Identified in number of studies (% of studies)
**Generic instruments used**	***Out of 22 identified studies***
EQ-5D-3/5L	8/22 (36%)
KPSS	2/22 (9%)
**Cancer generic instruments used**	
EORTC QLQ-C30	8/22 (36%)
**Indication-specific instruments used**	***Out of varying indication-specific identified studies***
***Lung cancer drugs***	
EORTC QLQ-LC13	4/6 (67%)
LCSS	1/6 (17%)
***Renal Cell Carcinoma drugs***	
FKSI-DRS	5/5 (100%)
FKSI-19	1/5 (20%)
FKSI-15	2/5 (40%)
***Ovarian cancer drugs***	
FACT-O	4/4 (100%)
***Breast cancer drugs***	
EORTC QLQ-BR23	1/6 (17%)
FACT-B	3/6 (50%)
**Type of analysis**	
Mean magnitude of change from baseline	17/22 (77%)
Time to worsening	9/22 (41%)
Proportion of responders	4/22 (18%)
**Reporting and analysis of missing data**	
Reported proportion missing at each measurement time	6/22 (27%)
Reported proportion missing post-baseline for some measurements	11/22 (50%)
Missing data adjustments (statistical analysis)	2/22 (9%)
**Protocol use**	
Protocol published	7/22 (23%)
All study instruments defined in clinical trial registration	9/22 (32%)

*Notes* EQ-5D-3 L/5L = EuroQol 5-Dimension with 3 or 5 Levels, KPSS = Karnofsky Performance Status Scale, EORTC QLQ-C30 = The EORTC Quality of Life Group Core Questionnaire, EORTC QLQ-LC13 = The EORTC Quality of Life Group Lung Cancer Questionnaire, LCSS = Lung Cancer Symptom Scale, FKSI-DRS/19/15 = Functional Assessment of Cancer Therapy Kidney Cancer Symptom Index Disease Related Symptoms/19-item version/15-item version, FACT-O = Functional Assessment of Cancer Therapy– Ovarian, EORTC QLQ-B23 = The EORTC Quality of Life Group Breast Cancer, FACT-B = Functional Assessment of Cancer Therapy– Breast

The most commonly used instruments for generic and cancer-generic QoL assessments were EQ-5D-3 L/5L and EORTC QLQ-C30 (used in 8 of 22 studies, respectively). In addition, the Karnofsky Performance Status Scale (KPSS) was used in 2 of the included studies. Of the indication-specific instruments, EORTC QLQ-LC13 was frequently used in lung cancer (4 of 6 studies). For renal cell carcinoma, the Functional Assessment of Cancer Therapy-Kidney Symptom Index was used in all identified studies (FKSI-DRS), as was the Functional Assessment of Cancer Therapy–Ovarian (FACT-O) in the identified studies on ovarian cancer. In breast cancer, EORTC-B23 was identified in 1 out of 6 studies and FACT-B in 3 out of 6 studies.

Table 1 also shows the number of studies with pre-registered trial protocols, including QoL measurement and analysis information. Nine studies had listed the QoL instruments in their pre-registration on Clinicaltrials.gov, and seven studies had published a study protocol or statistical analysis plan, including the QoL instruments and analyses (before manuscript submission).

The frequency of measurements differed to some extent between the included studies, and generally, no specific motivation was reported for the frequency of measurement. Several trials assessed QoL on the first day of each 28-day treatment cycle for the first few cycles and then less frequently in later cycles. Most studies finalized assessment at progression or treatment discontinuation, whereas a few studies continued QoL assessment in progressed states (details in Table 2).

**Table 2:** Extended summary of findings

Study & year	Instrument(s)	Score(s) used	Frequency of measurements (after baseline)	Statistical analyses	Reporting proportion missing data (% missing at 12 months^a^)	Missing data adjustments	Study protocol published	Instruments listed in registration
**Alectinib (NSCLC)**								
Pérol et al. 2019 [43]	QLQ-C30 QLQ-LC13	Average of raw item scores and global health status scores transformed to 0-100 range	Every 4 weeks until progression, at one post-treatment-visit and every 8 weeks up to 6 months after treatment discontinuation.	- Mean magnitude of change in score from baseline: not reported if and what type of statistical model was used to evaluate between-treatment differences. - Proportion of responders (proportion of patients improving, stable, deteriorating): not reported if and what type of statistical model was used to evaluate between-treatment differences. - Time to worsening ($\ge$10-point decrease at two consecutive assessments or followed by death within 5 weeks) using Kaplan-Meier and Log-rank test	Yes, per each measurement (27–38%)	No	Yes	Yes
Li et al. 2021 [44]	KPSS	0-100 range	Every 3 months until end of study follow-up.	- Proportion of responders (proportion of stable patients at last follow-up based on improving score or $\le$10-point deterioration from baseline): between-treatment comparison using chi-square test.	No	No	No	No
**Ceritinib (NSCLC)**								
Shaw et al. 2017 [45]	QLQ-C30 QLQ-LC13 EQ-5D-5 L LCSS	- Single domain/item scores - Summary/global scores (Eq. 5D tariff not defined, QLQ-C30)	First day of cycle two & three, whereafter every second cycle up until month 18, followed by every 9 weeks to treatment discontinuation.	- Mean magnitude of change in domain and summary scores from baseline (all instruments): between-treatment comparison using a longitudinal repeated measures model - Time to worsening for QLQ-LC13 & LCSS ($\ge$10-point decrease in any LC13 item and $\ge$15 mm increase in LCSS) using Kaplan-Meier methods and Cox regression.	Yes, per each measurement (17–25%)	No	No	No
**Osimertinib** **(NSCLC)**								
Leighl et al. 2020 [46]	QLQ-C30 QLQ-LC13	Average of raw item scores and global health status scores (range 0-100)	QLC-C30: Every 6 weeks QLC-LC13: Every week for 6 weeks, and then every 3 weeks. Length of follow-up not explicitly defined.	- Mean magnitude of change from baseline in QLQ-C30 score: between-treatment comparisons based on mixed-effect models with defined set of covariates - Proportion of responders (proportion of patients with a decrease (improvement) in a score by $\ge$10 points compared to baseline: between-treatment comparisons based on logistic regression - Time to worsening ($\ge$10 points difference compared to baseline on five pre-specified symptoms): between-treatment comparisons based on Kaplan-Meier methods	Yes, per each measurement (1–28%)	No	No	Yes
Lee et al. 2018 [47]	QLQ-C30 QLQ-LC13	Average of raw item scores and global health status scores (range 0-100)	C30: Every 6 weeks until study end or progression L13: Every week for 6 weeks, and then every 3 weeks until progression.	- Proportion of responders (proportion of patients with a decrease (improvement) in a score by $\ge$10 points compared to baseline: between-treatment comparisons based on logistic regression with defined set of covariates - Time to worsening ($\ge$10 points difference compared to baseline on five pre-specified symptoms): between-treatment comparisons based on log-rank test - Mean magnitude of change from baseline in QLQ-C30 score: between-treatment comparisons based on mixed-effect models with defined set of covariates	Yes, per each measurement (33–41%)	No	Yes	Yes
Nie et al. 2018 [48]	Undefined	Not reported	Not reported	Not reported	No	No	No	No
**Axitinib** **(RCC)**								
Motzer et al. 2013 [49]	FKSI-15 FKSI-DRS	Summary score (FKSI-15 range 0–60, FKSI-DSR range 0–36)	First day of every cycle until treatment discontinuation.	- Mean magnitude of change from baseline: between-treatment comparisons based on a mixed model with repeated measurements.	No	No	No	No
Qin et al. 2015 [50]	FKSI-15 FKSI-DRS EQ-5D	Summary score (FKSI-15 range 0–60, FKSI-DSR range 0–36, Eq. 5D score or tariff not defined)	Every 4 weeks until 28 days after treatment discontinuation.	- Mean magnitude of change from baseline: between-treatment comparisons based on a mixed model with repeated measurements. - Time to worsening for first event of FKSI-15 ($\ge 5$ point decrease), FKSI-DRS ($\ge$3 point decrease), progression or death using undefined survival analysis method.	Yes, while on treatment (up to 10% missing)	No	No	No
**Everolimus** **(RCC)**								
Beaumont et al. 2011 [42]	QLQ-C30 FKSI-DRS	Summary scores (FKSI-DRS range 0–36, QLQ-C30 global QoL and physical functioning scales range 0-100)	First day of every cycle until treatment discontinuation.	- Mean magnitude of change from baseline: between-treatment comparisons based on a mixed model with repeated measurements.	Yes, per some measurements (Not reported)	Pattern mixture models with patients stratified by missing data pattern	No	No
Cella et al. 2018 [51]	FKSI-19 FKSI-DRS EQ-5D-5 L	- Summary score (FKSI-19 range 0–78, FKSI-DSR range 0–36) and four subscales (varying ranges) - Index score (Eq. 5D)	Every 4 weeks until week 25, whereafter every 8 weeks until 52 weeks, whereafter every 12 weeks until last tumor imaging assessment.	- Mean magnitude of change from baseline in total scores, subscale and index score: between-treatment comparison based on a repeated measures model with defined covariates. - Time to worsening for FKSI-DRS ($\ge$4 point decrease) using Kaplan-Meier methods and Cox regression.	Yes, per some assessments (2–25% ^b^)	Sensitivity analyses by analyzing different dropout tertiles	No	No
Motzer et al. 2010 [52]	KPSS FKSI-DRS	- Score from 0 to 100 (KPSS) - Total score 0 to 36 (FKSI-DRS)	Day 1 of each cycle until treatment discontinuation	- Time to worsening ($\ge$10-point decrease in KPSS, ($\ge$2 points in FKI-DRS): using Kaplan-Meier methods and Log-rank test.	No	No	No	No
**Olaparib (Ovarian cancer)**								
Friedlander et al. 2018 [53]	FACT-O EQ-5D-5 L	- FACT-O TOI index range 0 to 100 - EQ-5D-5 L index score (range 0–1)	At week 5, week 13, and then every 12 weeks up until at most 24 months or data cut-off (incl. at treatment discontinuation and 30 days after discontinuation)	- Mean magnitude of change from baseline in TOI index: between-treatment comparisons based on mixed models for repeated measures - Quality-adjusted progression free survival (QAPS): mean EQ-5D index score multiplied by progression free survival with between-treatment comparisons evaluated by bootstrapping - Time without symptoms of treatment toxicity (TWiST): Time until treatment toxicity with between-treatment comparison evaluated by Kaplan-Meier methods.	Yes, per each measurement (8–9% ^c^)	No	No	No
Penson et al. 2020 [54]	FACT-O	- FACT-O TOI index range 0 to 100	At day 29, and then every 8 weeks up until week 48	- Mean magnitude of change from baseline in TOI index: between-treatment comparisons based on linear mixed-effect model with defined set of covariates.	Yes, per one measurement only (20–40% ^b^)	No	Yes	Yes
Liu et al. 2022 [55]	FACT-O EQ-5D	- FACT-O NFOSI-DRS-P (range 0–36) - Eq. 5D results not reported	Every 12 weeks for 3 years until treatment discontinuation.	- Mean magnitude of change from baseline in NFSOI-DRS-P: between-treatment comparisons based on mixed-effects model with defined set of covariates.	No	No	Yes	Yes
**Zejula (Ovarian cancer)**								
Oza et al. 2018 [56]	FACT-O EQ-5D	- FACT-O FOSI (range 0–32) - Eq. 5D-index score and VAS score	Every 8 weeks for first 14 cycles, every 12 weeks thereafter until treatment discontinuation,	- Mean magnitude of change from baseline in FOSI and Eq. 5D scores: between-treatment comparisons based on mixed-effect models with defined covariates (all Eq. 5D scores were averaged to assess one post-baseline score).	Yes, per some assessments (4–16% ^d^)	No	Yes	No^e^
**Fulvestrant** **(BC)**								
Robertson et al. 2018 [57]	FACT-B	- Total scores (FACT-B 0-144, FACT-B TOI 0–92)	Every 12 weeks until treatment discontinuation	- Mean magnitude of change from baseline in each score: between-treatment comparisons based on mixed-effect models with defined set of covariates. - Time to worsening ($\ge$8 points FACT-B sum and $\ge$6 points TOI score difference): between-treatment comparisons based on log-rank test and Kaplan-Meier method.	Yes, per most measurements (approx. 10–15%).	No	No	No^f^
**Kisqali (BC)**								
Janni et al. 2018 [58]	QLQ-C30	Global health status score (range 0-100)	Every 8 weeks for the first 18 months, then every 12 weeks until progression.	- Mean magnitude of change from baseline: between-treatment comparisons based on linear effect model.	No	No	No	Yes
**Palbociclib** **(BC)**								
Rugo et al. 2019 [59]	FACT-B	- Total scores (FACT-G, FACT-B, TOI; varying ranges) - Subscale scores (Physical, Social, Emotional, Functional, Breast Cancer)	At day 1 of cycle 2, 3, 5 and then every other cycle until progression or treatment discontinuation.	- Mean magnitude of change from baseline in each score: between-treatment comparisons based on mixed-effect models with defined set of covariates	No	No	No	Yes
Martin et al. 2021 [60]	QLQ-C30 QLQ-B23 EQ-5D-3 L	- QLQ C30 Global health status (0 to 100) - Results from other instruments not reported in study	Every 2 cycles for the first 7 cycles, then every 3 cycles until treatment discontinuation (and during one post-treatment visit)	- Time to worsening ($\ge$10-point decrease): between-treatment comparison based on Cox regression	Single estimate for 1 year completion provided (18%)	No	No	No
Xu et al. 2022 [61]	FACT-B EQ-5D-3 L	- Total scores (FACT-G, FACT-B, TOI; varying ranges) - Subscale scores (Physical, Social, Emotional, Functional, Breast Cancer) - Eq. 5D mentioned in text, but not reported in table/figure	Day 1 of cycle 2 and 3, and then day 1 of every other cycle until treatment discontinuation (last assessment on end of treatment visit).	- Mean magnitude of change from baseline in total and subscale scores: between-treatment comparisons based on mixed-effect models with undefined covariates included.	No	No	Yes	Yes
**Venclyxto (CLL)**								
Al-Sawaf et al. 2021 [62]	QLQ-C30	Average of raw item scores and global health status scores (range 0-100)	First day of each cycle until cycle 12, followed by at 1, 3, 6, 12, 15, 18, 24, and 30 months after treatment discontinuation.	- Mean magnitude of change from baseline in total and subscale scores: between-treatment comparisons based on mixed-effect models with defined covariates. - Proportion of responders (proportion of patients with a decrease (improvement) in a score by $\ge$10 points compared to baseline: between-treatment comparisons based on logistic regression with defined set of covariates	Yes, per some assessments (15% ^g^)	No	Yes	Yes
**Tafinlar (melanoma)**								
Grob et al. 2014 [63]	QLQ-C30	- QLQ C30 Global health status and symptom scales and single items (0 to 100)	At weeks 6, 12, 15, at progression and finally 4 weeks after progression.	- Mean magnitude of change from baseline in total and subscale scores: between-treatment comparisons based on mixed-effect models with defined covariates included.	No	No	No	No

*Notes *^a^ Share of missing data is the % of missing from the remaining eligible population at 1 year (i.e., complete patient dropout not included and any patients not completing baseline HRQoL also excluded from the nominator). ^b^ At 48 weeks. ^c^ At 49 weeks. ^d^ At the 4th cycle follow-up. ^e^ One of two instruments listed before submission. ^f^ Instrument included after final data collection, but before submission. ^g^ At 18-month follow-up. EQ-5D-3 L/5L = EuroQol 5-Dimension with 3 or 5 Levels, KPSS = Karnofsky Performance Status Scale, EORTC QLQ-C30 = The EORTC Quality of Life Group Core Questionnaire, EORTC QLQ-LC13 = The EORTC Quality of Life Group Lung Cancer Questionnaire, LCSS = Lung Cancer Symptom Scale, FKSI-DRS/19/15 = Functional Assessment of Cancer Therapy Kidney Cancer Symptom Index Disease Related Symptoms/19-item version/15-item version, FACT-O = Functional Assessment of Cancer Therapy– Ovarian, EORTC QLQ-B23 = The EORTC Quality of Life Group Breast Cancer, FACT-B = Functional Assessment of Cancer Therapy– Breast

The statistical analyses differed depending on the instrument and scoring system used. Most frequently, the analyses assessed between-group differences in the mean magnitude of change from baseline using longitudinal mixed-effect models (17 of 22 studies). The second most common analytical approach was to analyze the time to worsening with Kaplan-Meier and/or Cox regression methods (9 of 22 studies). Worsening in QLQ-C30 was consistently defined as a reduction by 10 points or more compared to the baseline score, but for other instruments, the definition of worsening varied (see Table 2). Finally, 4 of 22 studies analyzed the proportion of responders, similar to time to worsening, but typically based on assessing the proportion of patients not having worsened (i.e., maintaining or improving QoL) at a specific follow-up time. The proportion of responders analyses is thus typically modeled using binary outcome models (such as logistic regression).

Regarding missing data, some studies (6 of 22) reported the proportion missing for all measurement points (typically as supplementary material). In contrast, another set of studies reported the proportion of missing observations for a few specific time points (11 of 22), whereas five studies provided no data on the proportion of missing responses. Among the studies that reported missing data, the proportion of missing responses at 12 months follow-up (or close to 12 months) varied between 2 and 40%. The proportion of missing data was based on calculations where patients not responding at baseline and patients dropping out of the study due to disease progression were not included in the denominator. Despite the prevalent missing data problem, only two studies used a statistical approach to address the impact of missingness. The approaches to assessing the impact of missing data included pattern-mixture models and stratified analyses in sub-groups with varying missing data patterns. The full details of instruments used, frequency of assessment, and statistical analyses are shown in Table 2.

## Discussion

We reviewed QoL measurements and statistical analyses applied in published RCTs after reimbursement for cancer drugs that were initially reimbursed and introduced in Swedish healthcare with a lack of evidence of QoL and OS benefits. We identified any new publications from the original pivotal trial and any other trials for the same patient indication. Considering the increasing share of reimbursements based on surrogate endpoints and single-arm trials, it is essential to assess what type of robust evidence becomes available in the post-reimbursement period to confirm claims of clinical benefit– and that any QoL data becoming available is based on valid measures and analyses of QoL [31]. Out of 22 cancer drug indications reimbursed with limited evidence, we identified and reviewed RCTs for 12 drugs in 22 published studies. EORTC QLC-C30 [15] and EQ-5D-3/5L [14] were the most frequently used instruments. Both these instruments have previously been reported as the most commonly used instruments in cancer trials [23, 27, 32], and some studies have shown success in mapping QoL scores between these instruments [33, 34]. In addition, indication-specific instruments that were used included, e.g., EORTC QLC-LC13 (in 4 out of 6 lung cancer studies), FKSI-DRS (used in 5 out of 5 studies on renal cell carcinoma), and FACT-O (used in 4 out of 4 studies on ovarian cancer), and FACT-B (used in 3 out of 6 studies on breast cancer).

The US Food and Drug Administration (FDA) and the European Medicines Agency (EMA) encourage the assessment of QoL in cancer RCTs. In addition, an increasing number of payers consider QoL to be a valuable input to reimbursement and coverage decisions [35], and QoL data is a necessary input in cost-effectiveness analyses using QALYs as a health outcome measure. However, QoL data must be measured and analyzed validly and reliably to provide valuable insights to decision-makers and clinicians. The FDA has hesitated to grant QoL labeling of the product’s benefits, which may be attributed to uncertainties and a lack of quality in the measurement and analytical standards for QoL data [35, 36]. There are several calls for standardization of design, reporting, and statistical analyses of QoL data, including initiatives by the, e.g., SPIRIT-PRO [37], CONSORT-PRO [29], and SISAQOL [30] consortiums.

Our review highlights several relevant aspects with room for improvement. We found that several studies lacked information on the scoring system used, which, particularly for the EQ-5D and the range of available tariffs to predict QoL scores, can substantially impact the interpretation and comparability of results [38]. The frequency of measurements varied across studies and was rarely explicitly motivated– some studies assessed QoL every cycle (28 days), whereas other studies had three-month intervals. There may be well-motivated reasons for variations in the frequency of assessment between disease and study contexts, but this is difficult to identify when the rationale is not outlined. The approach for the timing of the last assessment also varied between studies. Some studies assessed QoL until disease progression and treatment discontinuation. In contrast, others assessed QoL until death or the last study follow-up, and in some studies, we could not identify information on the procedure for the timing of the final assessment. For QoL evidence to inform treatment decisions, capturing QoL consequences after disease progression is essential, particularly in settings where overall survival benefits have not been demonstrated [39]. For proper value assessments and cost-effectiveness analyses of drugs, it is also necessary to have valid QoL data both in the progression-free and progressed disease states.

Regarding the statistical analyses of the data, most studies used approaches that align with recommendations from, e.g., SISAQOL [30], including linear mixed-effects models for repeated measurements to assess between-treatment group differences in mean changes from baseline. However, many studies failed to report all relevant modeling choices, such as which covariates were included as control variables or whether variables (including time indicators) were treated as random or fixed effects. Besides including baseline QoL, additional covariates associated with the QoL outcome are often recommended to include to improve precision and power. Such covariates should be pre-defined in the study protocol or statistical analysis plan [40]. Based on our comparisons to clinical trial registrations and study protocols (including any statistical analysis plan referenced), only 9 of 22 studies listed all instruments analyzed in the study in the clinical trial registration before final data collection. Only seven had published their study protocol and included information for QoL analysis. Given the researcher’s degrees of freedom of choice involved in the measurement and analysis of QoL data, it is essential to have proper pre-registered documentation to increase the validity of the findings [41]. With the finding that most studies lacked appropriate pre-registration and study protocols, the QoL findings in these papers should primarily be interpreted as exploratory.

Finally, we found that many reviewed studies had substantial missing data. Among the studies that reported the share of missing data, it varied from only a few percent up to about 40%. However, the data on missingness generally excluded patients who did not complete baseline QoL assessments and those who declined follow-up in the study. Thus, the missing data shares can be considered conservative lower-bound estimates. In addition, despite the substantial missing data issue, only 2 out of 22 studies addressed the potential impact of missing data. This is a lower share than some earlier reviews of missing QoL data in cancer trials [25]. One of the studies addressing the missing data problem showed that missingness was associated with adverse QoL, implying that it is not reasonable to assume that data are missing at random [42]. The SISAQOL Consortium guidelines on handling missing data include nine items, e.g., that statistical approaches to assess missing data should be pre-specified in the protocol or statistical analysis plan and that at least two sensitivity analyses should be used [30]. Our review shows that missingness is an area where development is needed in QoL studies.

Our study has limitations that are important for interpretation and generalizability. First, our sample of included studies is based on identified published studies on cancer drugs for which there were no mature OS data or QoL data at the time of reimbursement. While our sample of studies thus reflects a practically relevant context where reimbursement decisions for costly drugs have been made based on limited evidence, the review cannot necessarily be interpreted as representative of the broader cancer drug trial literature. Second, it should be mentioned that several of the papers included in this review were published before recommendations for reporting and analysis by the SPIRIT-PRO extension (2018) and the SISAQOL Consortium Guidelines (2020). Thus, the papers included in this review should not be judged in terms of adherence to such reporting guidelines per se. Instead, we have used items from these guidelines as a proxy for what can be seen as best-practice reporting and analysis standards. Third, our study covers a subset of the criteria for best practices in the measurement and analyses of QoL data as outlined in the abovementioned guidelines. For example, this review does not assess whether the studies were explicit about a confirmatory or exploratory research objective, if any adjustments were considered for multiple hypothesis testing, or the type of potential arguments provided for the magnitude of missing data.

## Conclusion

QoL data is increasingly used to inform regulatory decisions and facilitate more patient-centered care. For QoL data to serve as input to improved patient outcomes and decision-making, QoL data must be measured and analyzed using appropriate methods. We documented several deviations from high-quality measurement and analysis standards for clinical trials based on reviews of post-reimbursement studies for cancer drugs initially reimbursed in Swedish healthcare with limited evidence of QoL or OS benefits. Our results suggest areas of improvement in QoL assessments related to handling missing data, motivations for the measurement frequency, and pre-trial protocol registration and adherence. Given the increasing focus on patient-reported outcomes and the use of QoL data, future QoL assessments from cancer drug trials must be conducted with stricter adherence to best-practice guidelines to provide valuable input to patient care and decision-makers.

### Electronic supplementary material

Below is the link to the electronic supplementary material.

Supplementary Material 1

## Data Availability

All data generated or analyzed during this study are included in this published article and its supplementary information files.
