# Supplementary material for: Analyses of quality of life in cancer drug trials - a review of measurements and analytical choices in post-reimbursement studies
Source: BMC Cancer. 2024 Mar 6;24:311. doi: 10.1186/s12885-024-12045-8 (PMC10916053; doi:10.1186/s12885-024-12045-8)
Supplement: Supplementary file 1 — Supplementary Material 1 [file 12885_2024_12045_MOESM1_ESM.docx]

**SUPPLEMENTARY MATERIAL**

“Analyses of Quality of Life in Cancer Drug Trials - A Review of Measurements and Analytical Choices in Post-Reimbursement Studies”

Table of Contents

[Search Strategy ii](#_Toc149038580)

[Figure S1. Flow diagram iii](#_Toc149038581)

[Table S1. Cancer drug indications with identified post-reimbursement QoL data iv](#_Toc149038582)

# Search Strategy

We collaborated with university librarians to develop the search strategy. We used the search terms *“active substance name OR drug brand name AND cancer form AND Cochrane Highly Sensitive Search Strategy for identifying randomized trials”* for each of the 22 drug-indications approved by TLV between 2010 and 2020 with no statistically significant data on QoL or OS at the time of reimbursement. The search was also conducted by including the search term synonyms from the National Institutes of Health cancer dictionary and the Library of Medical Subject Heading (MESH) terms.

The time period for each drug indication was set to one year before the TLV reimbursement decision up to October 2022 (when the last search was conducted). The choice of allowing the inclusion of papers published before the TLV decision was made to capture potential studies that may have been published just before (or during) the final reimbursement process and thus not necessarily included in the evidence dossier handled by TLV.

Inclusion criteria were that studies in English or Swedish had to report QoL data from an RCT with the patient population criteria set to match the reimbursed population per the TLV decision. All authors of this manuscript collaborated on the screening and selection of studies (at least two authors independently evaluated each identified abstract and, later, full-text). We refer to Chauca Strand et al 2023 (1) for the complete search terms and results for each drug indication.

# Figure S1. Flow diagram


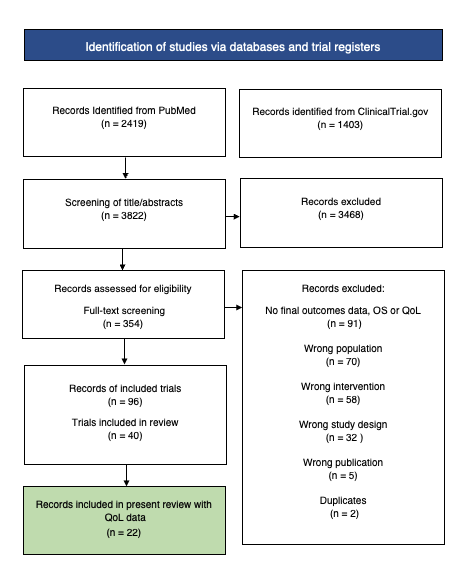


**Note:** Adapted from Chauca Strand et al 2023 (28), under Creative Commons license <http://creativecommons.org/licenses/by-nc/4.0/>.

# Table S1. Cancer drug indications with/without identified post-reimbursement QoL data

| **Drug** | **Indication** | **Identified QoL evidence post-reimbursement (Number of studies)** |
| --- | --- | --- |
| Afinitor  (Everolimus) | Patients with advanced RCC, following VEGFRtargeted therapy treatment. | Yes (3) |
| Tasigna  (Nilotinib) | Newly diagnosed patients with Ph+ CML. | No |
| Inlyta  (Axitinib) | Patients with advanced RCC after failure of prior treatments with sunitinib or cytokines. | Yes (2) |
| Adcetris  (Brentuximab Vedotin) | Patients with recurrent or refractory CD30+ Hodgkin’s Lymphoma; following ASCT or following at least two prior therapies when ASCT or chemotherapy is not a treatment option or will be doing a ASCT (used as neo-adjuvant treatment). | No |
| Bosulif  (Bosutinib) | Patients with Ph+ CML in chronic, accelerated or blast phase previously treated with one or more TKIs and for whom imatinib, nilotinib and dasatinib are not considered appropriate treatment options. | No |
| Tafinlar  (Dabrafenib) | Patients with unresectable or metastatic melanoma with a BRAF V600 mutation. | Yes (1) |
| Cabometyx (Cabozantinib) | Patients with progressive, unresectable locally advanced or metastatic medullary thyroid carcinoma | No |
| Lynparza  (Olaparib) | Maintenance treatment of patients with platinumsensitive relapsed high-grade epithelial ovarian, fallopian tube, or primary peritoneal cancer with BRCA-mutations who are in response to platinum-based chemotherapy. | Yes (3) |
| Zydelig  (Idelalisib) | Patient with follicular lymphoma that is refractory to two previous treatments. | No |
| Iclusig  (Ponatinib) | Patients with CML in chronic, accelerated or blast phase, who are resistant to dasatinib or nilotinib; or who are intolerant to dasatinib or nilotinib and for whom subsequent treatment with imatinib is not appropriate; or who have a T315I mutation. | No |
| Iclusig  (Ponatinib) | Patients with Ph+ Acute lymphocytic leukemia who are resistant to dasatinib and for whom subsequent treatment with imatinib is not appropriate; or who have T315I mutation. | No |
| Zykadia  (Ceritinib) | Patients with advanced ALK+ non-small cell lung cancer who previously have been treated with crizotinib. | Yes (1) |
| Ibrance  (Palbociclib) | Patients with locally advanced or metastatic HR+, HER2- breast cancer (in combination with an aromatase inhibitor). | Yes (3) |
| Tagrisso  (Osimertinib) | Patients with locally advanced or metastatic nonsmall cell lung cancer with EGFR T790Mmutation. | Yes (3) |
| Alecensa  (Alectinib) | Patients with ALK+ advanced non-small cell lung cancer who have been previously treated with crizotinib. | No |
| Alecensa  (Alectinib) | As first-line treatment for patients with ALK+ advanced, non-small cell lung cancer. | Yes (2) |
| Faslodex  (Fulvestrant) | Patients with ER+ locally advanced or metastatic breast cancer in postmenopausal women not previously treated with endocrine therapy. | Yes (1) |
| Kisqali  (Ribociclib) | In combination with an aromatase inhibitor as initial endocrine-based therapy for postmenopausal women with locally advanced or metastatic HR+, HER- breast cancer. | Yes (1) |
| Lorviqua  (Lorlatinib) | Patients with ALK+ advanced non-small cell lung cancer whose disease has progressed after treatment with alectinib or certinib as first ALK tyrosine kinase inhibitor therapy; or crizotonib and at least another ALK TKI. | No |
| Zejula  (Niraparib) | As maintenance treatment for patients with platinum-sensitive relapsed high-grade serous epithelial ovarian, fallopian tube or primary peritoneal cancer who are in response after platinum-based chemotherapy. | Yes (1) |
| Venclyxto (Venetoclax) | In combination with obinutuzumab for the treatment of adults with previously untreated CLL. | Yes (1) |
| Vitrakvi  (Larotrectinib) | Treatment of patients with solid tumors with NTRK-gene fusion with locally advanced or metastatic disease or where surgical resection is likely to result in severe morbidity or who have no satisfactory treatment options. | No |

**Notes:** Abbreviations: ALK+, anaplastic lymphoma kinase-positive; ASCT, autologous stem cell transplantation; CML, chronic myelogenous leukemia; CLL, chronic lymphocytic leukemia; HR+, hormone-receptor positive; HER2, human epidermal growth factor receptor 2; NTRK, Neurotrophic tyrosine receptor kinase; OS, overall survival; PFS, progression-free survival; Ph+, Philadelphia chromosome-positive; QoL, quality of life; RCC, renal cell carcinoma; TKI, tyrosine-kinase inhibitor. Table Adapted from Chauca Strand et al 2023 (28), under Creative Commons license <http://creativecommons.org/licenses/by-nc/4.0/>.
